# Analysis of Historical Sources of Heavy Metals in Lake Taihu Based on the Positive Matrix Factorization Model

**DOI:** 10.3390/ijerph15071540

**Published:** 2018-07-20

**Authors:** Yan Li, Liping Mei, Shenglu Zhou, Zhenyi Jia, Junxiao Wang, Baojie Li, Chunhui Wang, Shaohua Wu

**Affiliations:** 1 School of Geographic and Oceanographic Sciences, Nanjing University, 163 Xianlin Road, Nanjing 210023, Jiangsu, China; dg1627013@smail.nju.edu.cn (Y.L.); zhenyijay@smail.nju.edu.cn (Z.J.); dz1427034@smail.nju.edu.cn (J.W.); baojieli@smail.nju.edu.cn (B.L.); ch_wang1987@163.com (C.W.); wsh@nju.edu.cn (S.W.); 2 Key Laboratory of Coastal Zone Exploitation and Protection, Ministry of Land and Resources, Nanjing 210008, Jiangsu, China; 3 School of Chemistry and Chemical Engineering, Nanjing University, 163 Xianlin Road, Nanjing 210023, Jiangsu, China; dg1624048@smail.nju.edu.cn

**Keywords:** sediment, positive matrix factorization, heavy metal, source resolution

## Abstract

Analysis of sediment grain sizes and heavy metal correlations in the western part of Lake Taihu shows that the grain size of the sediment is stable as a whole. With increasing depth, the grain size tends to decrease. Heavy metals such as Cr, Cd, Pd and Sr are strongly correlated and influence each other. Based on the positive matrix factorization (PMF) model, this study classified the origin of heavy metals in the sediments of western Lake Taihu into three major categories: Agricultural, industrial and geogenic. The contributions of the three heavy metal sources in each sample were analyzed and calculated. Overall, prior to the Chinese economic reform, the study area mainly practiced agriculture. The sources of heavy metals in the sediments were mostly of agricultural and geogenic origin, and remained relatively stable with contribution rates of 44.07 ± 11.84% (*n* = 30) and 35.67 ± 11.70% (*n* = 30), respectively. After the reform and opening up of China, as the economy experienced rapid development, industry and agriculture became the main sources of heavy metals in sediments, accounting for 56.99 ± 15.73% (*n* = 15) and 31.22 ± 14.31% (*n* = 15), respectively. The PMF model is convenient and efficient, and a good method to determine the origin of heavy metals in sediments.

## 1. Introduction

Lake Taihu, located in Southern Jiangsu Province, is the third largest freshwater lake in China, with an area of 2338 km^2^, and plays a decisive role in regional and socioeconomic development. Heavy metals enter aquatic systems via surface runoff or atmospheric deposition and accumulate in sediments through the adsorption and sedimentation of suspended matter. Heavy metals are important pollutants of aquatic ecosystems because of their environmental persistence, toxicity and potential for accumulation in food chains [1,2,3,4]. Lake Taihu water flows in mainly from the west, and the environmental quality of the western part of the lake plays a crucial role in the environment of the entire lake. However, the sources and history of heavy metals in the sediments of West Lake Taihu are unclear. Therefore, it is necessary to investigate these issues and provide a basis for the monitoring and management of heavy metal pollution.

At this stage, the models of source determination for sediment heavy metals can be divided into two main types: diffusion and receptor-oriented models. The diffusion model (which involves a mathematical model combined with certain assumptions to select a series of parameters to calculate the diffusion and migration of pollutants in a simulation of the actual situation), has the pollution source as the main research object [5,6]. The diffusion equation can be used to calculate the source contributions according to the pollutant discharge, distance between the study area and the discharge source, physical and chemical properties of the pollutants, discharge rate, weather conditions, and other parameters. On the other hand, the receptor-oriented model (a digital model and method for identifying and analyzing the different sources and contribution rates of receptor contaminants) takes a contaminated area as the research object [7,8,9,10]. The positive matrix factorization (PMF) model is one of the source resolution methods recommended by the US Environmental Protection Agency [11] (USEPA). The method is mainly applied to air pollution, water pollution, sediment, etc. [7,12,13] and has been shown to be a powerful model for analyzing the source of material in the environment [13,14].

In this study, we investigated the heavy metal concentrations in the sediment of Lake Taihu. The specific objectives were as follows: (1) to analyze characteristics of the grain size of Lake Taihu sediments; (2) to measure the concentrations of heavy metals in the sediments; and (3) to analyze the sources of heavy metals in the sediments of western Lake Taihu and quantify the historical changes in the sources of heavy metals in the sediments using the PMF model.

## 2. Materials and Methods

### 2.1. Study Area and Sampling

**Figure 1:**
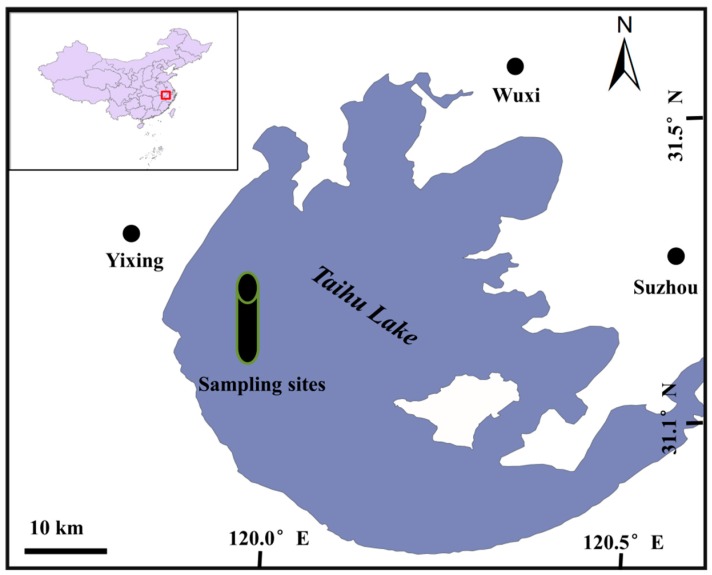
Location of the study area and sampling sites.

Lake Taihu Basin is a key area in eastern China because of its high population. Rainfall in the area, controlled by the Pacific monsoon, is moderately high (905–1956 mm/a), and precipitation in summer is approximately 37% of the total annual rainfall. The average annual evaporation is approximately 1001 mm, and the mean annual temperature is 16 °C. In this study, we selected the western part of Lake Taihu, shown in Figure 1, and collected a 45-cm long sediment column using a non-interfering gravity sampler. The column was sliced into 1 cm samples (a total of 45 samples) and freeze-dried at −40 °C for 48–72 h.

### 2.2. Analysis of Sediment

A laser grain size analyzer (Malvern Mastersizer 2000) was used to measure the grain size (a separate sample aliquot of 2 g of sediment was pretreated using 15 mL of 0.6% (NaPO_3_)_6_ for 24 h to be dispersed and homogenized). The total heavy metal concentrations and ^210^Pb in the prepared samples were determined with a HCl-HNO_3_-HF-HClO_4_ extraction [15]. Approximately 100 mg of sample was digested with 3 mL of 37% HCL, 1 mL of 65% HNO_3_, 6 mL of 65% HF, and 0.5 mL of 65% HClO_4_. There were two steps as follows: in the first step, the temperature was increased to 200 °C in 15 min, and in the second step it was maintained at 200 °C for approximately 20 min to continue digestion. The sample digestion solutions were evaporated to near dryness after the microwave pretreatment and then dissolved with 65% HNO_3_, after which 20 mL of deionized water was added to each sample. Prior to the experimental analysis, the solution was stored in 25-mL high-density polyethylene vials. The concentrations of K, As, Mn, Cu, Fe, Zn, Al, Ni, Sr, Ti, Ni and Cr were determined by inductively coupled plasma optical emission spectrometry (ICP-OES), and the concentrations of Cd and Pb were determined with an inductively coupled plasma mass spectrometer (ICP-MS). The accuracy and precision were verified using standard reference materials (soil GBW07405, Chinese geological reference materials). In addition, blank and duplicate samples were also prepared and analyzed (Tables S1 and S2), and the detection limit, quantification limit and recovery rate are shown in Table S4.

The activity of ^210^Pb was determined by γ analysis, using a high-purity germanium detector (EC & GORTEC, Meriden, CT, USA), digital spectrometer and multi-channel analysis system. The sample measurement time was 40,000 s, with a measurement error controlled at the 95% confidence level and measured data errors of less than 5%.

### 2.3. Data Analysis

The Al is the main component of clay minerals, and its content is an important indicator of the coarse grain size. It is an ideal element to normalize the effect of grain size on the content of heavy metals. In this study, Al was used to normalize heavy metal elements [16,17,18], using the following formula:

(1) $$C_{N}^{i}=C_{0}^{i}/C_{O}^{Al}$$

where $C_{N}^{i}$ is the normalized value of the *i* element in the sediment; $C_{0}^{i}$ is the measured value of the *i* element in the sediment; $C_{O}^{Al}$ is the measured value of the Al in the sediment [19].

PMF multivariate statistical analysis was proposed by Paatero and Tapper [20]. PMF is applied mainly through the least squares method to determine the source of the receptor media and its contribution rate. Its mathematical principle is expressed as follows: define matrix X, which can be expressed as *n* × *m*, where *n* represents the number of samples and *m* represents the number of contaminants; decompose matrix X into two factor matrices and one residual matrix, G (*n* × *p*), F (*p* × *m*) and E (*n* × *m*), respectively, where *p* is the number of pollution sources; the mathematical formula is expressed as X = GF + E; the matrix F represents the factor loading, the matrix G represents the factor contribution, and the matrix E represents the residual matrix, defined as the difference between the actual data and the result of the analysis.

The matrix conversion is performed as follows:

(2) $$X_{ij}=\sum_{k=1}^{p}g_{ik}f_{kj}+e_{ij}$$

where X*_ij_* represents the content (concentration) of the *j*th pollutant in the *i*th sample; *g_ik_* represents the contribution rate of the *k*th source to the *i*th sample; *f_kj_* represents the content of the *j*th pollutant in the source *k* concentration; *e_ij_* represents the residual matrix. At the same time, PMF defines an objective function:

(3) $$Q(E)=\sum_{i=1}^{n}\sum_{j=1}^{m}{\left(\frac{e_{ij}}{u_{ij}}\right)}^{2}$$

where *u_ij_* denotes the *j*th pollutant uncertainty of the *i*th sample. The uncertainty is calculated based mainly on the uncertainty of the sample measurement (MU) and the method detection limit (MDL). When the sample content (concentration) is less than or equal to the MDL, the uncertainty [21] *u* is calculated as:

(4) $$u=\frac{5}{6}\times \mathrm{MDL}$$

When the sample content (concentration) is greater than the MDL, the uncertainty *u* is calculated as:

(5) $$u=\sqrt{{\left(MU\times concentration\right)}^{2}+{\left(MDL\right)}^{2}}$$

PMF analysis was performed using the USEPA PMF 5.0 model (USEPA, 2014). The IBM SPSS Statistics 21 package was used for data description and correlation analysis.

## 3. Results and Discussion

### 3.1. Sediment Properties

**Figure 2:**
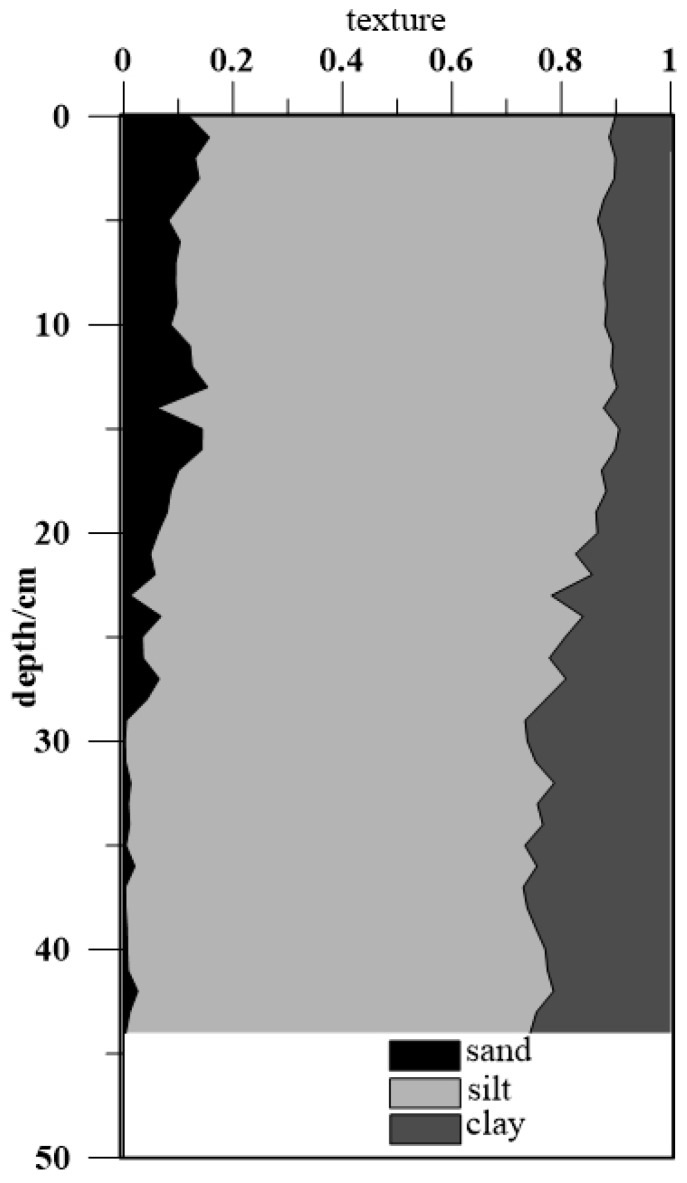
Changes of sediment grain size with depth.

Figure 2 shows the variation in particle size with depth in the sedimentary column. Most samples could be classified as sandy silt, clayey silt, or silty sand [22]. In the sediment column, the silt fraction was predominant, and its proportion varied between 73% and 82% with an average content of 76%. The proportion of the clay fraction varied between 9% and 27% with an average content of 17%. The sand fraction of the sediment samples gradually increased from the bottom to the surface, and the variation range was 0–16%. In general, the particle size in the sediment column tended to increase from the bottom to the surface and remained relatively stable as a whole, indicating that the sediment column particle diameter had not been affected by historical floods [23,24].

### 3.2. ^210^Pb Geochronology

**Figure 3:**
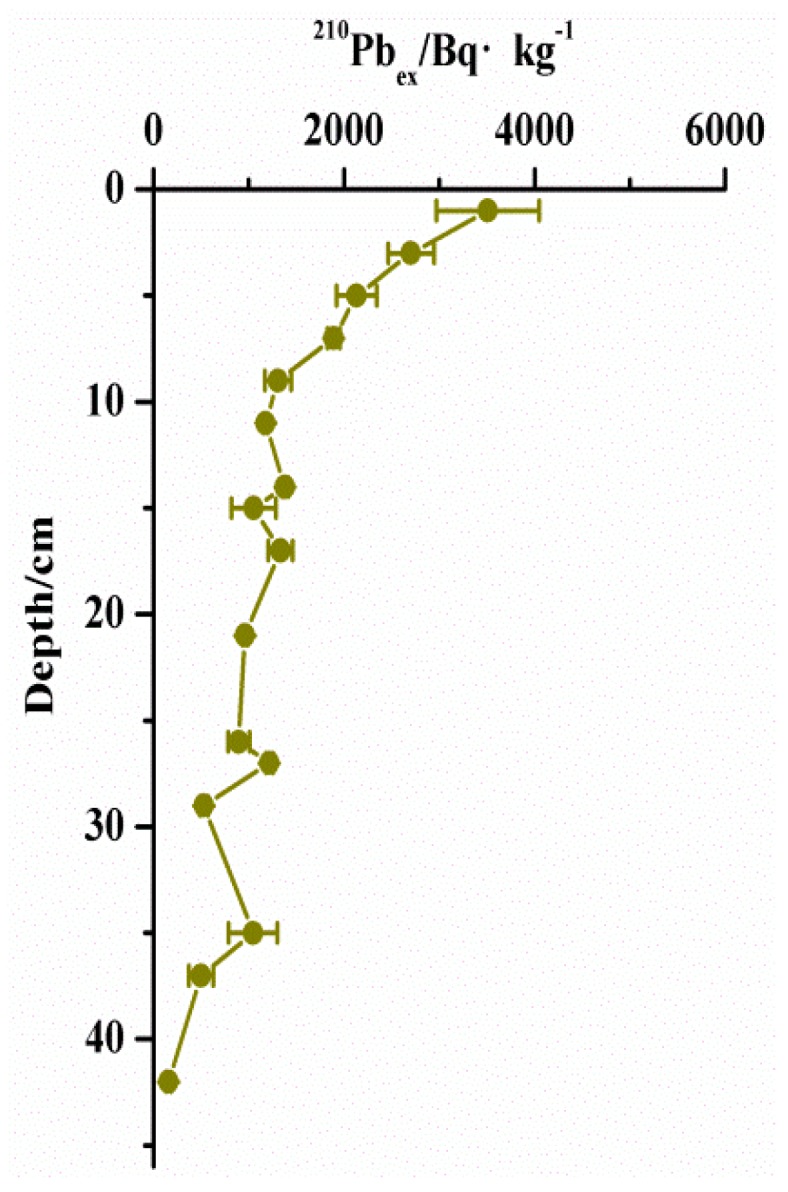
Vertical profiles of ^210^Pb_ex_ in Lake Taihu.

The profiles of excessive ^210^Pb activity in the sediment core are shown in Figure 3. The vertical profiles of the excessive ^210^Pb activity in the sediment core exhibited an approximately monotonic decline with depth. The ^210^Pb_ex_ values in the core were significantly correlated with sample depths (*R*^2^ = 0.765), which indicates that the slices from both of the cores can reflect historic change. The constant initial concentration (CIC) mode of ^210^Pb requires a better index distribution of the ^210^Pb_ex_ specific activity with depth, so the study area with a stable depositional environment tends to use the CIC mode to calculate the year [25,26]. In this study, the dates were calculated based on a CIC of ^210^Pb model [25,26].

### 3.3. Heavy Metal Characteristics

On the basis of the dating, the description and analysis of the heavy metals in the upper part of the sediment column (after 1978 ± 0.7838) and the lower part (before 1978 ± 0.7838) showed that the content of heavy metals such as Cr, Cd, Sr and Pb were obviously higher in the upper part than in the lower part. In Table 1, the average concentrations of Cr and Cd in the upper part were 70.6 mg kg^−1^ and 465.1 μg kg^−1^, respectively; in the lower part, they were 51.7 mg kg ^−1^ and 136.0 μg kg^−1^, respectively. The coefficients of variation for the other elements were not significantly large, and the coefficients of variation for various metals after the 1980s were slightly larger than those before the 1980s. Correlation analysis (CA) was performed.

Table 2 lists the correlation coefficients between the deposition parameters (clay, silt, sand) and metals (Cr, Cu, Fe, Mg, Mn, Ni, K, Sr, Ti, Zn, Cd, Pb, Al and As). The CA of the data showed that heavy metals such as Cu, Fe, Mg and Al were positively correlated with the clay fraction and negatively correlated with the sand fraction (significant at *p* < 0.01), reflecting the adsorption and enrichment of heavy metals on fine clay [27,28]. However, Cr, Cd, Pd, Sr and other heavy metals were negatively correlated with fine clay (significant at *p* < 0.01), and there was no correlation for Ti and Fe. With the decrease of fine grain content, these heavy metal contents increased, reflecting the impact of human activities on the import of heavy metals into lake sediments [29,30].

### 3.4. Source Identification by PMF

We used PMF to analyze the sources of heavy metals in the sediments of the western Lake Taihu. The PMF model was simulated with three to six factors, and the starting point of each run was different. We selected a random seed pattern with 20 random origins and checked for three to six factors. Finally, the PMF analysis identified three appropriate factors in the sediments of the western Lake Taihu (the uncertainty of the factor changed by 10%, and its analysis results are shown in Table S3; the analysis of the model with four to six factors is shown in Table S5). The tentative identification of the factor profiles was based on the characteristic geochemical signature from different sources.

**Figure 4:**
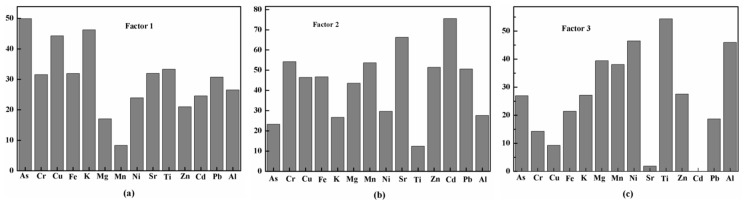
Source profiles obtained from the positive matrix factorization (PMF) model. (**a**) Factor 1 agricultural sources; (**b**) Factor 2 industrial sources; (**c**) Factor 3 geogenic sources.

The source composition of the three-factor solution is summarized in Figure 4, with factor 1 accounting for 29% of the total heavy metals in the sediments of western Lake Taihu, dominated by As, K and Cu. According to previous studies, fertilization can effectively increase the concentration of K in the soil [31,32]. Elements such as As and Cu are commonly used to make pesticides [33]. The main agricultural practice in the study area is the growing of rice and tea. To increase output, local farmers often use pesticides and chemical fertilizers such as sodium methylarsonate and potassium [34]. Therefore, factor 1 indicated agricultural sources. Factor 2 had a degree of interpretation of 46% and was dominated by Cd, Cr, Pb, Sr and Mn. The research area mainly has metallurgical industry, building materials industry, ceramic industry and so on [34,35]. These industries can produce large quantities of pollutants containing Cd, Cr, Pb and Mn in the process of production [36,37,38]; thus, factor 2 represented industrial sources. Factor 3 accounted for 25% of the total heavy metals and was mainly composed of Ti, Al and Ni, while Mg also showed some loadings on this factor. Ti, Al and Ni mainly come from the erosion of the riverbed and dissolution of minerals; thus, factor 3 represented geogenic sources [39].

**Figure 5:**
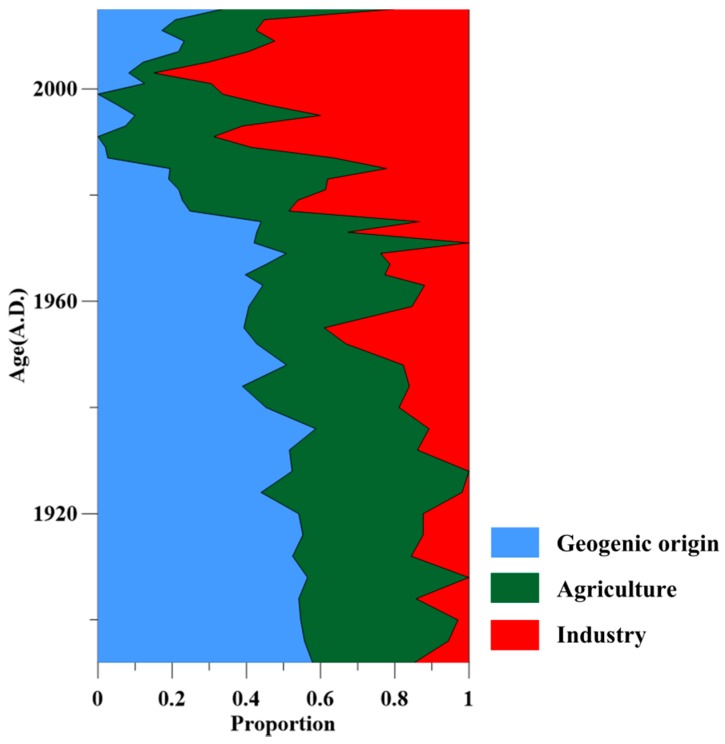
Variation in the composition of pollution sources of sediment heavy metals with time based on the positive matrix factorization (PMF) model.

**Figure 6:**
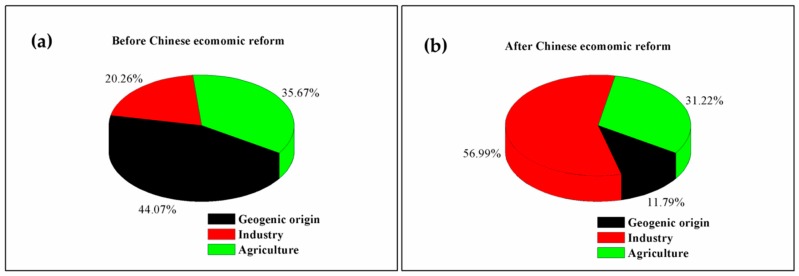
Sources of sediment heavy metals before and after the Chinese economic reform. (**a**) before Chinese economic reform; (**b**) after Chinese economic reform.

The PMF model was successfully applied to the identification of sediment pollution sources in western Lake Taihu. Figure 5 shows that before the 1980s, the contributions of industry, agriculture and geological processes to the origin of heavy metals were relatively stable, with a significant contribution of geological processes and agriculture, and a certain degree of contribution of industry. After the 1980s, the contribution of geological processes to sediment heavy metals decreased rapidly, while that of industry rose rapidly and reached stability in approximately 2003. Before the reform and opening up of China, the contributions of industry, agriculture and geological processes to sediment heavy metals were 20.26 ± 15.61% (*n* = 30), 35.67 ± 11.70% (*n* = 30) and 44.07 ± 11.84% (*n* = 30), respectively. After the reform and opening up, their contributions to heavy metals were 56.99 ± 15.73% (*n* = 15), 31.22 ± 14.31% (*n* = 15) and 11.79 ± 9.83% (*n* = 15), respectively (Figure 6a,b). In recent years, the proportion of heavy metals in sediments originating from industrial sources has declined.

**Figure 7:**
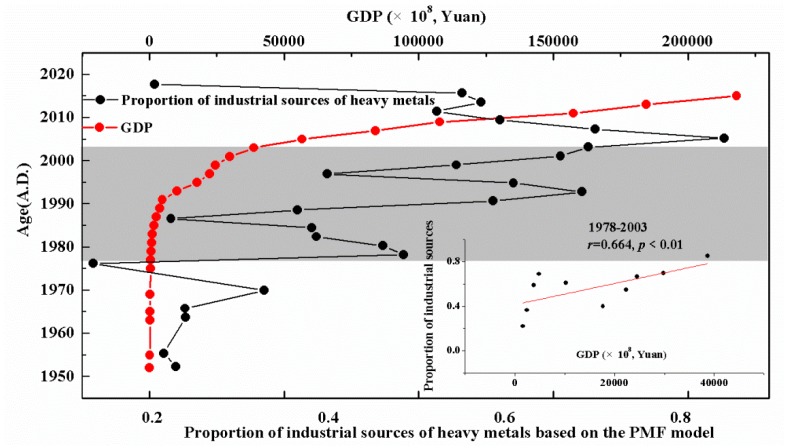
Correlation analysis of the proportion of industrial sources of heavy metals based on the PMF model and the GDP of the study area.

This trend is in good agreement with historical policies and economic development in China. Figure 7 shows the relationship between the GDP of Jiangsu Province (Jiangsu Statistical Yearbook, 1975–2015) [40] and the ratio of industrial sources of heavy metals based on the PMF model. Before 2003, there was a significant positive correlation between the GDP of Jiangsu Province and ratio of industrial sources of heavy metals. With the rapid increase in GDP, the proportion of heavy metals produced by industrial activities rose quickly. However, after 2003, the two variables showed opposite trends, and economic development did not increase the proportion of heavy metals produced by industrial activities. This phenomenon has also been reported in the records of lakes and marine sediments in other regions [41,42,43]. For example, Li [11] and Wan [44] reported that the pollutants in Chaohu and Gonghai reached their maximum level in the late 20th century and began to decline in the early 21st century. These research results are in agreement with ours and indicate that the PMF model in the present study is highly reliable.

Before the 1980s, China’s scientific, technological and economic conditions were limited, with agriculture as the mainstay. After the reform and opening up in 1978, China’s economy developed rapidly, and the output value of industry and agriculture soared [40]. At the same time, this development brought about serious environmental pollution problems. The proportion of heavy metal pollution caused by industrial production was highest in the early 21st century. With the promulgation and implementation of a series of environmental laws, China’s environment has improved greatly after the 2000s. The proportion of heavy metal pollution brought about by industrial production has been curbed and is showing a downward trend, as shown in Figure 5.

## 4. Conclusions

With the development of the Chinese economy, heavy metal pollution became increasingly serious. In general, this period can be divided into two stages. Before 1978 ± 0.7838, the heavy metals in the study area were mainly of geogenic and agricultural origins. After 1978 ± 0.7838, rapid industrial development occurred, and the heavy metals in the sediments originated mainly from industry and agriculture and were less affected by geological processes. This result is in good agreement with the GDP of the study area. The PMF model and quantitative analysis of sediment for heavy metal sources from the bottom depths to the top provide an efficient and convenient combination of analytical methods.

## Figures and Tables

**Table 1:** Heavy metals in sedimentary columns around 1978 (± 0.7838).

Upper Part (After 1978 ± 0.7838)					Lower Part (Before 1978 ± 0.7838)			
	Minimum	Maximum	Mean	CV	Minimum	Maximum	Mean	CV
Cr (mg kg^−1^)	51.3	84.0	70.6	14.9	43.1	67.8	51.9	10.8
Cu (mg kg^−1^)	13.5	27.8	19.7	24.2	17.6	36.2	23.9	18.1
Fe (mg kg^−1^)	22,284.0	27,289.9	24,972.3	5.3	22,747.9	28,965.8	25,869.8	6.3
Mg (mg kg^−1^)	5286.6	7783.3	6407.2	11.0	5996.2	9648.3	8216.9	11.2
Mn (mg kg^−1^)	504.2	1231.0	810.7	27.1	487.6	1044.2	811.9	20.4
Ni (mg kg^−1^)	30.6	92.9	42.5	39.4	34.4	47.1	41.1	8.8
K (mg kg^−1^)	15,576.8	18,928.6	17,341.9	5.9	15,240.7	21,698.1	18,935.6	9.7
Sr (mg kg^−1^)	131.4	268.5	198.6	19.5	67.8	255.9	123.8	38.6
Ti (mg kg^−1^)	3984.0	5963.2	4492.8	9.8	3894.2	5128.1	4374.5	7.0
Zn (mg kg^−1^)	89.2	153.8	114.7	17.7	94.4	129.2	110.9	9.0
Cd (mg kg^−1^)	0.125	0.836	0.465	61.8	0.082	0.244	0.136	28.5
Pb (mg kg^−1^)	17.4	29.4	23.6	17.2	18.1	23.7	20.8	7.6
Al (mg kg^−1^)	22.0	34.9	27.0	11.8	21.5	43.6	35.0	15.4
As (mg kg^−1^)	16.3	33.6	21.5	18.1	14.9	43.5	25.5	27.8

CV: coefficient of variation in %.

**Table 2:** Pearson’s correlation of metal concentrations for Lake Taihu sedimentary column.

	Cr	Cu	Fe	Mg	Mn	Ni	K	Sr	Ti	Zn	Cd	Pb	Al	As	Sand	Silt	Clay
Cr	1																
Cu	−0.057	1															
Fe	−0.166	0.286	1														
Mg	−0.487 **	0.691 **	0.594 **	1													
Mn	0.264	0.669 **	0.282	0.490 **	1												
Ni	0.343 *	0.477 **	0.180	0.224	0.435 **	1											
K	−0.208	0.629 **	0.599 **	0.863 **	0.570 **	0.277	1										
Sr	0.557 **	−0.213	0.087	−0.227	0.387 **	0.194	0.073	1									
Ti	0.188	−0.254	−0.013	−0.257	−0.452 **	0.006	−0.153	−0.163	1								
Zn	0.437 **	0.709 **	0.355 *	0.364 *	0.814 **	0.600 **	0.475 **	0.357 *	−0.163	1							
Cd	0.801 **	0.140	−0.116	−0.373 *	0.457 **	0.472 **	−0.191	0.562 **	−0.083	0.659 **	1						
Pb	0.652 **	0.343 *	0.083	−0.092	0.570 **	0.487 **	0.077	0.335 *	−0.092	0.772 **	0.861 **	1					
Al	−0.526 **	0.349 *	−0.127	0.451 **	0.060	−0.030	0.254	−0.597 **	−0.104	−0.176	−0.504 **	−0.278	1				
As	−0.247	0.191	0.477 **	0.293	−0.011	0.070	0.216	−0.127	−0.039	0.179	−0.065	0.023	−0.043	1			
Sand	0.599 **	−0.507 **	−0.340 *	−0.766 **	−0.030	−0.058	−0.556 **	0.652 **	0.049	0.043	0.646 **	0.361 *	−0.639 **	−0.246	1		
Silt	0.260	−0.468 **	−0.186	−0.410 **	−0.181	−0.081	−0.267	0.433 **	0.344 *	−0.122	0.181	−0.011	−0.311 *	−0.142	0.370 *	1	
Clay	−0.573 **	0.588 **	0.328 *	0.780 **	0.106	0.062	0.562 **	−0.677 **	−0.168	0.013	−0.588 **	−0.282	0.626 **	0.234	−0.943 **	−0.648 **	1

* Significantly correlated at the 0.05 level (2-tailed); ** Significantly correlated at the 0.01 level (2-tailed).

## Supplementary Materials

The following are available online at http://www.mdpi.com/1660-4601/15/7/1540/s1, Table S1: Operating parameters of ICP-MS (ELAN 9000, Perkin-Elmer SCIEX) for the determination of element concentrations, Table S2: Operating parameters of ICP-OES (Optima 5300DV, Perkin-Elmer SCIEX) for the determination of elemental concentrations, Table S3: Uncertainty analysis of the model, Table S4: Detection limit, Quantification limit and Recovery rate for the heavy metals, Table S5: Factor Profiles (% of factor total).
