# Supplementary material for: Analysis of Historical Sources of Heavy Metals in Lake Taihu Based on the Positive Matrix Factorization Model
Source: Int J Environ Res Public Health. 2018 Jul 20;15(7):1540. doi: 10.3390/ijerph15071540 (PMC6068659; doi:10.3390/ijerph15071540)
Supplement: Supplementary file 1 [file ijerph-15-01540-s001.pdf]

Table S1. Operating parameters of ICP-MS (ELAN 9000, Perkin-Elmer SCIEX) for the determination of element concentrations.

Table S2. Operating parameters of ICP-OES (Optima 5300DV, Perkin-Elmer SCIEX) for the determination of elemental concentrations

Table S3. Uncertainty analysis of the model

Table S4. Detection limit, Quantification limit and Recovery rate for the heavy metals

Table S5. Factor Profiles (% of factor total)

Table S1. Operating parameters of ICP-MS (ELAN 9000, Perkin-Elmer SCIEX) for the determination of element concentrations.

Parameter	Value	Parameter	Value
Nebulizer gas flow (L min^{-1})	0.94	RF power (W)	1100
Analog stage voltage (V)	-1700	Lens voltage (V)	6
Pulse stage voltage (V)	900	Ac rod offset (V)	-6
Discriminator threshold (V)	70	Scan mode	Peak hopping
Speed of peristaltic pump (rpm)	26	Detector	Pulse
Sweeps/Reading	50	Replicates	2
Sampler/Skimmer cones	Nickel	Dwell Time (ms)	2.5
Spray chamber	Ryton® Double-pass Scott-type spray chamber		
Nebulizer	Gem-tip Cross-Flow pneumatic nebulizer		

Table S2. Operating parameters of ICP-OES (Optima 5300DV, Perkin-Elmer SCIEX) for the determination of elemental concentrations.

Parameter	Value
**Plasma argon flow**	**15 L/min**
Auxiliary argon flow	0.2 L/min
Nebulizer argon flowm	0.8 L/min
Sample flow rate	1.50 mL/min
Spray chamber	Cyclonic spray chamber
Nebulizer	Concentric glass (Meinhard) nebulizer
Viewing position	(15, 0)

Table S3. Uncertainty analysis of the model.

Caption		CV	Mean	Maximum	Minimum	SD
Before Chinese ecomomic reform	Industry	0.08	20.96	22.9	17.9	1.73
	Agriculture	0.06	34.59	37.79	32.69	2.08
	Geogenic origin	0.02	44.45	45.98	42.29	1.05
After Chinese ecomomic reform	Industry	0.05	55.14	61.03	52.16	2.94
	Agriculture	0.12	33.14	37.11	26.16	4.01
	Geogenic origin	0.21	11.71	17.51	10.14	2.50

CV representation of coefficient of variation, SD is a standard deviation.

Table S4. Detection limit, Quantification limit and Recovery rate for the heavy metals.

	**As**	**Cr**	**Cu**	**Fe**	**K**	**Mg**	**Mn**	**Ni**	**Sr**	**Ti**	**Zn**	**Cd**	**Pb**	**Al**
Detection limit (μg g^{-1})	0.1	1.5	0.4	0.035	0.04	0.03	2.5	0.5	0.3	0.45	0.018	0.001	0.006	0.2
Quantification limit (μg g^{-1})	0.3	4.5	1.2	0.105	0.12	0.09	7.5	1.5	0.9	1.6	0.06	0.003	0.02	0.6
Recovery rate (%)	73.7	109.7	112.3	89.3	85.3	83.7	76.7	83.3	88.0	73.7	95.3	92.3	89.3	85.7

Table S5. Factor Profiles (% of factor total).

	**Four factors**				**Five factors**					**Six factors**					
	Factor 1	Factor 2	Factor 3	Factor 4	Factor 1	Factor 2	Factor 3	Factor 4	Factor 5	Factor 1	Factor 2	Factor 3	Factor 4	Factor 5	Factor 6
As	0.0	39.9	4.4	55.6	17.4	26.5	16.5	0.0	39.5	8.3	0.0	6.0	0.0	48.8	36.9
Cr	4.2	12.3	38.4	45.2	40.1	10.1	28.5	8.8	12.6	12.7	10.9	24.1	13.3	38.9	0.0
Cu	23.5	14.7	1.3	60.5	30.6	25.7	10.2	33.0	0.6	4.7	29.1	14.9	5.0	29.9	16.3
Fe	5.9	30.6	16.1	47.5	25.6	13.2	16.4	7.8	37.0	18.6	15.7	5.2	13.0	33.2	14.3
K	10.8	26.7	12.3	50.3	27.3	13.6	10.9	14.4	33.8	16.7	20.0	4.0	15.7	29.6	13.9
Mg	16.0	28.4	0.1	55.5	24.2	18.0	4.2	21.6	32.0	14.4	25.9	0.0	13.7	27.0	19.1
Mn	22.5	3.2	9.9	64.4	41.7	19.0	0.5	33.2	5.5	0.0	21.6	16.4	22.1	29.3	10.6
Ni	15.0	19.9	13.2	51.9	29.3	19.3	18.5	20.6	12.4	10.9	21.4	15.5	7.0	32.2	13.0
Sr	0.0	0.0	41.2	58.8	51.5	0.1	0.1	0.1	48.1	9.1	0.0	6.0	44.1	40.7	0.1
Ti	3.3	33.3	24.8	38.6	22.7	9.4	26.6	2.4	39.0	25.0	15.0	8.2	9.9	32.0	9.9
Zn	12.0	16.8	15.2	56.1	34.5	19.0	14.5	19.2	12.9	8.4	18.6	14.8	11.0	35.8	11.3
Cd	0.0	0.0	52.9	47.1	75.3	9.0	14.1	0.0	1.6	0.0	0.0	34.6	12.4	53.0	0.0
Pb	9.7	16.3	24.3	49.8	34.4	16.0	22.9	14.9	11.8	9.3	14.0	21.4	10.3	36.5	8.6
Al	24.6	30.8	15.7	29.0	12.6	5.0	24.0	20.9	37.4	19.5	17.2	17.1	19.2	8.4	18.5
